# Light-Triggered Inflation of Microdroplets

**DOI:** 10.1021/acs.chemmater.4c00732

**Published:** 2024-04-12

**Authors:** Adam W. Hauser, Qintian Zhou, Paul M. Chaikin, Stefano Sacanna

**Affiliations:** † Department of Chemistry, New York University, 29 Washington Place, New York, New York 10003, United States; ‡ Center for Soft Matter Research, Department of Physics, New York University, 726 Broadway Avenue, New York, New York 10003, United States

## Abstract

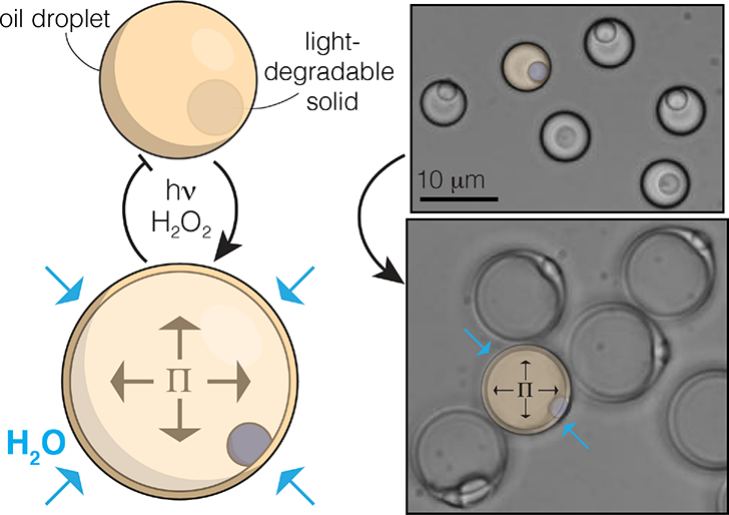

Driven systems composed largely of droplets and fuel make up a significant portion of microbiological function. At the micrometer scale, fully synthetic systems that perform an array of tasks within a uniform bulk are much more rare. In this work, we introduce an innovative design for solid-in-oil composite microdroplets. These microdroplets are engineered to nucleate an internal phase, undergo inflation, and eventually burst, all powered by a steady and uniform energy input. We show that by altering the background input, volumetric change and burst time can be tuned. When the inflated droplets release the inner contents, colloidal particles are shown to transiently attract to the release point. Lastly, we show that the system has the ability to perform multiple inflation–burst cycles. We anticipate that our conceptual design of internally powered microdroplets will catalyze further research into autonomous systems capable of intricate communication as well as inspire the development of advanced, responsive materials.

## Introduction

Synthetic responsive systems driven far from equilibrium are of interest for their potential insights into living matter^1−4^ and to provide tools for advanced multifunctional or autonomous materials.^5,6^ Living systems comprise a chorus of out of equilibrium subsystems that work to maintain homeostasis, where from one vantage point a chaotic flux of material and energy is exchanging and from another, a quiescence.^7^ Synthetic materials have yet to come near the complexity of their simplest living counterparts if we think of the full living material, but many interesting active or autonomously responsive “units” have been demonstrated.^8,9^ Increasing the diversity in scale, synthesis, materials, and response of such autonomous units remains a challenge to be addressed before a complex adaptive synthetic matter is to be proposed.

In synthetic active matter, there is a wealth of work focused on individual motility of so-called microrobots or the collective behavior of concentrated systems that often show emergent ordered phases.^10−15^ Less attention has been paid to simplified microscale units or cells designed for transient or delayed responses,^16−19^ or those that show potential for spatially and temporally localized information release^20−22^ - a common and necessary feature in biological function. Additionally, bottom-up synthetic methods are necessary for broad adoption and use in crowded or more complex systems.

**Figure 1:**
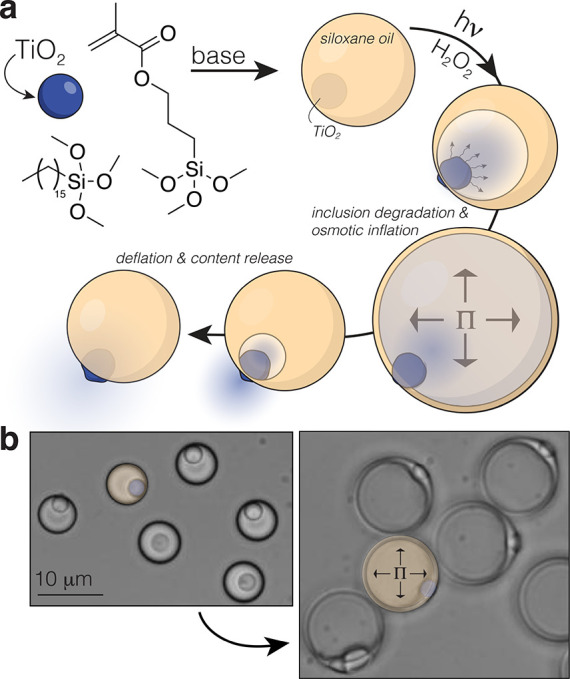
Light-triggered inflation summary. (a) TiO_2_ colloids are used as seeds for heterogeneous nucleation and growth of siloxane oil droplets. The TiO_2_ inclusion begins to degrade upon introduction of fuel (H_2_O_2_) and blue light, which in turn creates an osmotic pressure that initiates the inflation process until a failure occurs and the inner contents are released to the local surroundings; (b) optical images of droplets before and during inflation. Images are lightly false-colored to clarify the components to the reader: TiO_2_ is blue and siloxane oil is yellow.

In this work, we demonstrate that synthetic microscale solid-in-oil composite droplets can be triggered to nucleate an internal liquid phase, inflate themselves, and then burst all with constant and uniform energy input. This is achieved by encapsulating a self-degrading solid that releases contents in a semipermeable oil droplet which leads to an osmotic pressure, bringing in water from the bulk to inflate the newly formed internal aqueous phase until bursting (Figure 1). We find that the inflation rate and ultimate volume can be controlled by bulk conditions and light intensity due to an interplay of solid particle migration and degradation rate. We then show how this droplet design can be used to elicit transient responses in the bulk and lead to oscillatory behavior through a fundamentally new delayed release mechanism.

## Results and Discussion

Though one can imagine many material–reaction combinations with the potential to exhibit the described behavior, we exemplify the concept with a simple system consisting of a TiO_2_ colloid engulfed in a siloxane oil phase immersed in a degradative fuel (H_2_O_2_). Colloidally stable and light-degradable TiO_2_ colloids are synthesized by a modified method from Cao et al, where titanium(IV) isopropoxide is hydrolyzed in the presence of alkyl amines.^23^ Generally, these particles are heat-treated to remove organics and convert to the more catalytically active anatase form.^24^ In this case, we hydrothermally treat the particles for a short period of time (2–6 h) at 150 ^◦^C to impart some crystallinity but retain all the organic structure directing agent. The particles placed in basic hydrogen peroxide solution show a marked volume decrease with time when illuminated with blue light (Figure S3). Though the hydrothermal treatment step is not explicitly needed,^25,26^ we find that untreated particles tend to degrade much more rapidly in dark conditions. Thus, the alkyl amine and TiO_*x*_ species are the osmolytes, and the undegraded particle is the osmolyte reservoir that can be triggered to release contents with blue light.

To form the composite droplets, TiO_2_ colloids are used as seeds for heterogeneous nucleation and growth of the siloxane oil. Hexadecyltrimethoxysilane (HTMS) is first injected into a basic aqueous suspension of TiO_2_ to grow a hydrophobic layer, quickly followed by 3-(trimethoxysilyl)propyl methacrylate (TPM) that makes up the majority of the oil phase. Figure S1 shows that this bottom-up method provides size-uniform composite droplets with the solid particles completely encapsulated within the oil phase. Detailed synthetic procedures can be found in the Methods section.

**Figure 2:**
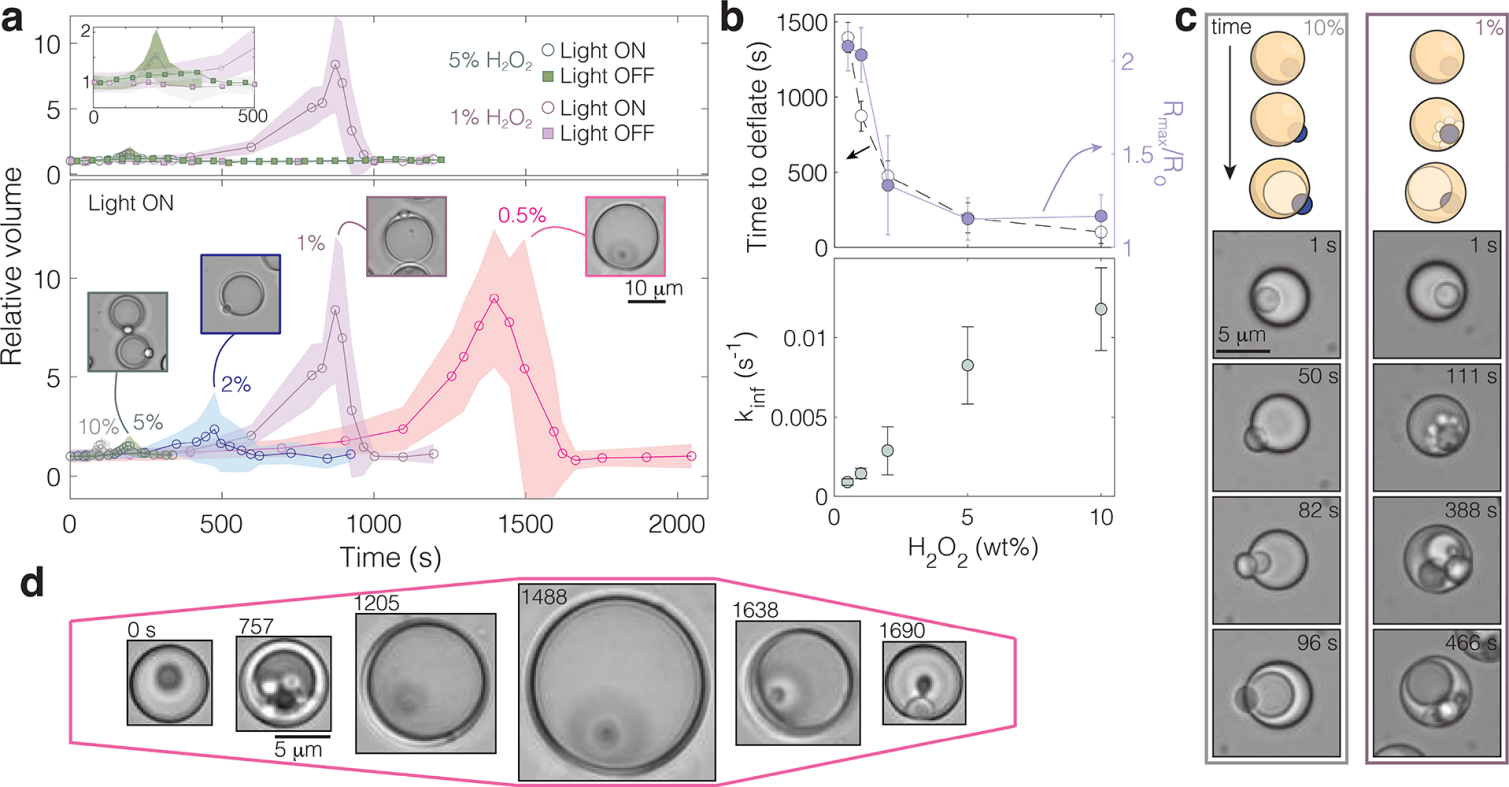
Bulk fuel affects inflation behavior. (a) (top) Average behavior of droplets at two fuel concentrations with the light on (open circles) or off (closed squares) indicating very little volumetric changes without light, and (bottom) various fuel concentrations with the light on showing a marked fuel effect; (b) average time to deflate (dashed line, white marker) and maximum size decrease with increasing fuel (solid line, purple marker), but inflation rate *k*_inf_, as determined by single exponential fits to the inflation portion of the data, increases; (c) schematics and data of single droplets at early times for 10 and 1 wt % H_2_O_2_ showing that the TiO_2_ tends to migrate before inflation at high fuel concentration and remains encapsulated when the fuel concentration is low; (d) representative images of a single droplet over the course of an inflation–deflation cycle with 0.5% H_2_O_2_.

We find that when these composite particles are placed in a basic H_2_O_2_ solution and illuminated uniformly with blue light, they remarkably nucleate a new aqueous internal phase and inflate until a bursting event occurs (Figure 2). The oil phase is permeable enough^27,28^ to allow water, NaOH and H_2_O_2_ to reach the surface of the TiO_2_ particle, but significantly less so to the degraded species. This sets up an osmotic pressure. In an ideal membrane, the inflation rate is primarily dictated by the osmolyte release rate, which approximates the degradation rate, so long as these rates are low enough that we are operating under pseudoequilibrium. If we explore the bulk fuel (H_2_O_2_) concentration while maintaining all else constant, we find that indeed the rate of inflation increases with fuel concentration as one might expect, but the ultimate inflated volume is inversely related (Figure 2a and b).

Intuitively, an osmotic pressure increase at a faster rate should not directly affect the ultimate volume. Taking a closer look at the inflation cycle reveals an apparent correlation between the average deflation time and the maximum size (Figure 2b). This leads us to examine the cycle at the single particle level. Figure 2c shows the early time behavior for both 10 and 1 wt % H_2_O_2_. At high fuel concentrations, the TiO_2_ first migrates out of the oil phase, creating new surface area exposed to the bulk surroundings, and then nucleates an internal phase before inflating. Lower fuel and thus lower inflation rates show several internal nucleation sites forming and coalescing during the course of inflation without any clear TiO_2_ migration. The former affords high inflation rates with small volume changes, while the latter gives low rates but large volume changes.

If a portion of the osmolyte reservoir releases into the bulk, it is clear that the resulting osmotic pressure will be lower than that of a fully encapsulated counterpart. As for the TiO_2_ migration, we surmise that conditions favoring fast degradation also have a higher surface-bound hydrophobic HTMS detachment rate, which triggers oil dewetting.^29^ Once an inner aqueous–solid interface is formed, the migration of TiO_2_ ceases and inflation occurs. Therefore, the rate ratio of surface HTMS detachment *k*_d_ to osmolyte release *k*_r_ determines the overall behavior in time. When *k*_d_/*k*_r_ is low, the osmolyte release and inner phase nucleation occur before the oil dewetting becomes favorable and thus, the TiO_2_ rarely migrates before inflation (Figure 2c). The nonuniform oil wall thickness further supports this; where, the thinnest portion resides at the oil-solid interface for high *k*_d_/*k*_r_ and the opposite occurs for low *k*_d_/*k*_r_. Moreover, if we decrease the fuel concentration to 0.1 wt %, we observe a majority of droplets contain large multiple compartments that resist coalescence, suggesting that the bound HTMS may still be sparsely present on the TiO_2_ surface (Figure S4).

We observe that both decreasing pH or light intensity affords less TiO_2_ migration, lower inflation rates and higher inflation volumes (Figure S2). This allows for increased complexity through external spatial and temporal control by light patterns or intensity modulation (Figure S7).

**Figure 3:**
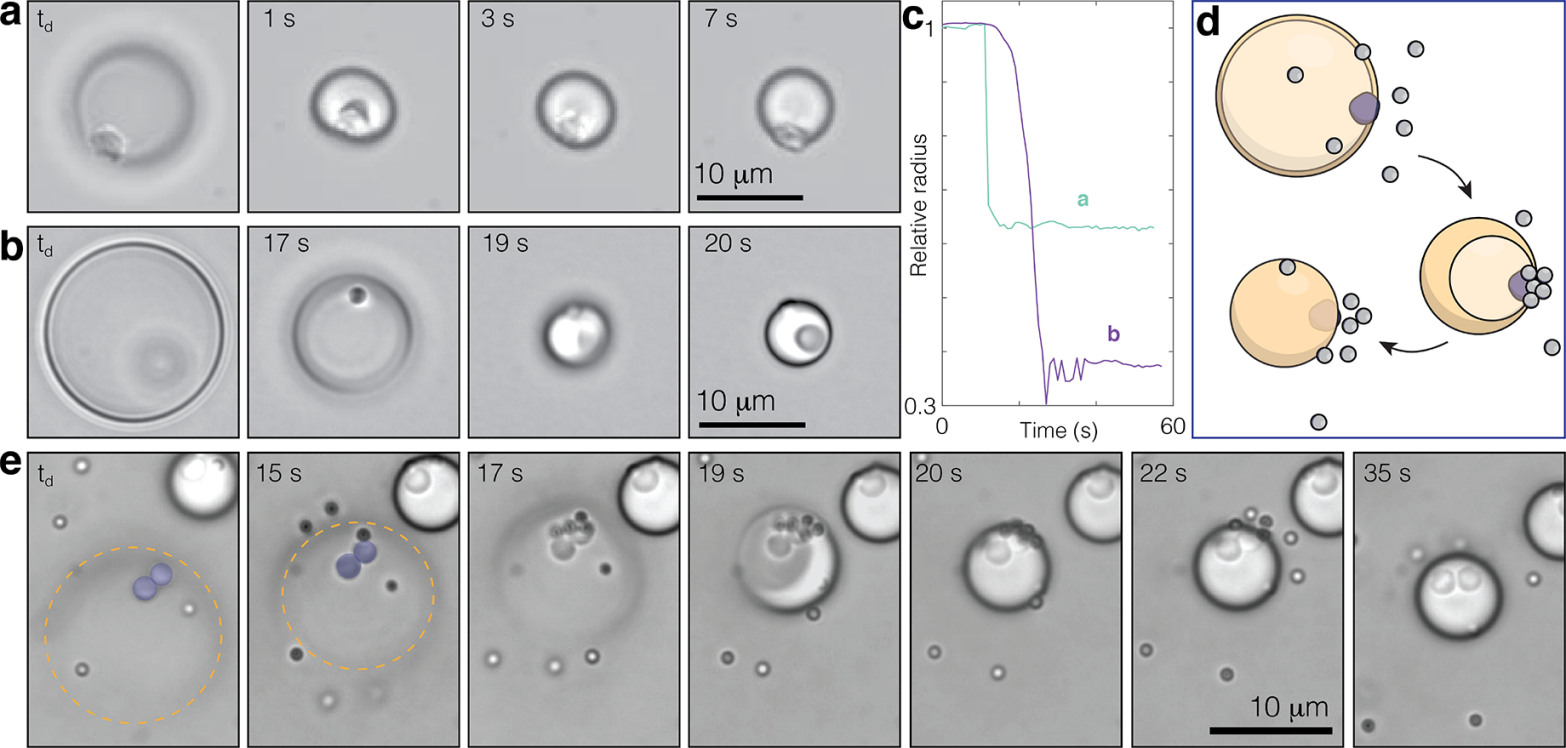
Burst-release of inner contents transiently attracts passive particles. Deflation tends to occur over the course of either about 1 or 10 s, as shown in (a) and (b), respectively; *t*_d_ is the time at which deflation begins; (c) relative radius of a representative fast and slow deflation event with time; schematic (d) and example (e) of a slow deflation and release of inner contents in the presence of colloidal tracers demonstrating a transient collection near the release point and a return to Brownian behavior once a gradient is no longer sensed; droplet outer diameter and TiO_2_ are indicated in color in the first two panels.

We then investigate the burst behavior at the single droplet level. The incitement and speed of burst events are of interest, as they will determine how the osmolyte is released to the local environment. We observe a fast burst in which contents are released in 1 s or less, or a slow release that occurs over 10–20 s (Figure 3a–c). The propensity of a slow release increases with a decreasing inflation rate, but some fast bursts still occur in these conditions. Regardless of the burst behavior, the incident failure point is located near the TiO_2_. If the entire inner phase is slowly released through a pore, we produce a highly localized chemical gradient that could affect the near-field bulk surroundings. To test this idea, we add small 800 nm particles that are colloidally stable in equilibrium with the composite droplets through charge repulsion and inflate the composite droplets at low inflation rates. Remarkably, tracers are seen rushing toward the release point and cluster there until the inner phase has released entirely, at which point the tracers no longer sense a chemical gradient and become Brownian once again (Figure 3d,e). This is a rare synthetic example where uniform bulk energy inputs afford a delayed and localized gradient release that elicits a transient response. We suspect the directed motion arises largely from diffusiophoresis^30^ of the tracer particles toward a higher concentration of degradant chemicals. Future studies will additionally explore various local detectors beyond simple colloids, which have the potential to elicit cascading interactions. We note that the deflating droplets exhibit self-driven motion during a deflation event. Though not exhaustively studied, the release appears to have a repulsion effect on the droplets; that is, droplets that slowly release contents tend to swim away from the failure point, and other droplets in the vicinity tend to increase their distance from a deflating droplet. This can be observed where noted in the Supporting Videos.

**Figure 4:**
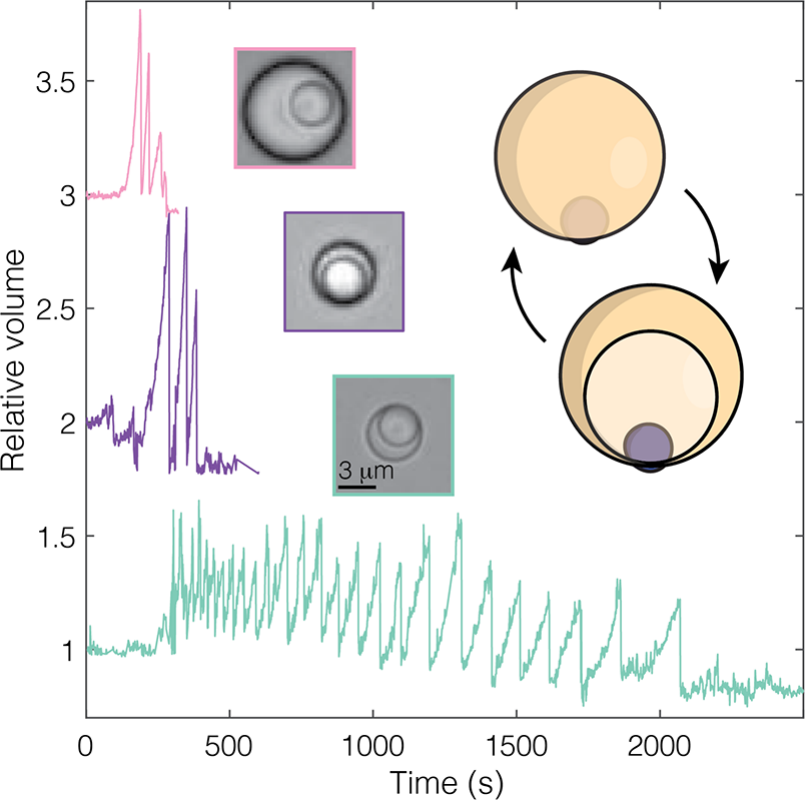
Reinflation cycling behavior. Relative volume with time for three representative droplets with different continuous bulk input that show reinflation behavior; (top) large oil/solid ratio used throughout this study at 5 wt % H_2_O_2_, and smaller oil/solid ratio droplets in 1% H_2_O_2_ using a trimethoxysilane TPM (middle) or dimethoxysilane DPM (bottom) showing that subtle changes in oil volume or chemistry can drastically affect reinflation.

Finally, we explore conditions under which droplets undergo cyclic inflation–burst cycles. As everything but our solid osmolyte reservoir is a liquid, we hypothesized that upon bursting, the liquids would repair and another inflation cycle would begin as long as the reservoir was not spent. With the droplets and conditions discussed above, this only occurs when the inflation rate is sufficiently high (≥5 wt % H_2_O_2_) resulting in 2–4 damped cycles as shown in Figure 4. By lowering the oil/solid volume ratio, we can induce slower oscillations with lower inflation rates but the cycle number remains less than 5. By replacing the trimethoxysilane with a dimethoxysilane under similar bulk inputs, 10–20 cycles can be achieved. We point to the interfacial tension and viscosity of the oil phase as drivers for rewetting post burst. The dimethoxysilane has a higher interfacial tension with the water phase and a lower viscosity, and if all else is equivalent this should increase the rewetting propensity after a burst relative to the trimethoxysilane counterpart. Permeation of reactants and products through the oil is another important factor when considering new oil phases. We believe that this is a step toward new self-regulating behavior without inherently oscillatory chemical reactions^31−33^ from very simple ingredients and bottom-up methods.

## Conclusion

We have presented a fully synthetic bottom-up scheme to synthesize solid-in-oil composite microdroplets that when given certain uniform bulk inputs nucleate an inner aqueous phase, inflate, and burst. The inflation cycle is tuned by bulk conditions and is shown to rely on the interplay between solid–oil dewetting and osmolyte release rates. Upon bursting, we show that individual droplets release a chemical gradient that can transiently attract nearby particles. Lastly, we show the potential of our system and systems like this to exhibit oscillatory inflation-burst behavior with unchanging bulk inputs. The materials presented here should by no means uniquely provide the responses shown; thus, we hope and expect following iterations to increase complexity and control to move toward synthetic communicating systems and advanced material applications.

## Experimental Section

### Materials

All chemicals were used as received unless otherwise noted. Titanium(IV) isopropoxide (TIP, 97%), hexadecylamine (HDA, 98%), anhydrous butanol, potassium chloride (>99%), hexadecyltrimethoxysilane (HTMS, > 85%), sodium hydroxide, hydrogen peroxide (30%), ammonium hydroxide (30% in water), Pluronic F108, potassium persulfate (KPS, > 99%), styrene (>99%) and 2-hydroxy-2-methylpropiophenone (97%) were purchased from Sigma-Aldrich. 3-(trimethoxysilyl)propyl methacrylate (TPM, 98%) and methacryloxypropylmethyldimethoxysilane (DPM, 92%) were purchased from Gelest inc. Absolute ethanol was purchased from Fisher Sci.

### Methods

#### TiO_2_ Colloids

The light-degradable TiO_2_ are synthesized according to methods by Cao et al.^23^ In a typical synthesis 300 mL of butanol is added to a 600 mL beaker, and 5.96 g HDA is added and stirred for about 45 min covered. 2.4 mL of 0.1 M KCl is then added to the mixture. 6.79 mL of TIP is injected quickly to the vigorously (700 rpm) stirring solution and allowed to continue stirring for 2 min, after which the solution is recovered and allowed to sit undisturbed for 18 h. The solution becomes very cloudy within 3 min of TIP introduction. 150–200 mL is decanted from the beaker, and the remaining suspension is centrifuged, decanted and dilute >10× with ethanol, and centrifuged again. The particles are washed with ethanol four more times before storing at about 10 wt % in ethanol. Centrifugation cycles were 5 min at 200 RCF. Before hydrothermal treatment, a portion is transferred to water by centrifugation. In a PTFE lined bomb reactor 15 mL of 2.5 wt % TiO_2_ in deionized water is added, sealed and placed in a 150 °C oven for 2 h. Heat treated colloids are washed three times with deionized water before use.

#### Composite Microdroplets

The hydrothermally treated TiO_2_ is used as seeds for heterogeneous nucleation and growth of siloxane oil. To alter the size, the silane to seed ratios are adjusted, while holding all other parameters constant. Sequential injection of HTMS and TPM was inspired by procedures from Aubret et al.^34^ The typical synthesis is as follows: to a 50 mL polypropylene tube, 25 mL deionized water, 100 μL of 3 wt % ammonium hydroxide, and 200 μL of hydrothermaly treated TiO_2_ at 10 wt % in water are added. 500 μL total silane is injected sequentially; first, 83 μL of HTMS followed by gentle swirling, then 417 μL of TPM followed by gentle swirling. The entire vessel is quickly taken to a rotating carousel (IKA Loopster) and rotated at 10 rpm for 60 min. The reaction is quenched by adding 250 μL of Pluronic F108 (5 wt % in water) then 50 μL of 1 M HCl, then immediately dilute to 50 mL, and washed by centrifugation and dilution with water at 100 RCF for 10 min three times. Droplets are stored in about 1 mL in the fridge and used within 24 h for inflation experiments. For DPM droplets, the same procedure is used substituting for TPM. To observe the droplets in SEM, the droplets can be UV-polymerized by the addition of 5 μL of 2-hydroxy-2-methylpropiophenone to 10 mL of a 10x diluted droplet stock and placed in a 365 nm UV reactor for 20 min.

The HTMS layer is not a requirement for the general phenomenon, but it does, however, decrease the surface detachment rate, which results in a greater range of behavior between bulk condition changes. Without the HTMS, the rate of surface detachment *k*_*d*_ increases and dewetting occurs more readily, resulting in lower overall volume changes and shorter inflation cycles. Other trimethoxysilane agents were explored in addition to HTMS, but water solubility and miscibility and reactivity differences between TPM and other compounds limited the success. As other oils are explored in similar systems, the additional primer and barrier layer may not be necessary.

#### Polystyrene Tracers

The polystyrene tracer colloids are synthesized by known surfactant-free emulsion polymerization methods. To synthesize 800 nm diameter particles, seeded growth using 400 nm seed particles is employed. For the seeds, 500 mL of deionized water and 50 mL of styrene is added to a 1 L 3-neck round-bottom flask. The mixture is brought to 60 ^◦^C, sparged with N_2_ and stirred for 30 min before injecting 0.21 g of KPS dissolved in 10 mL of deionized water. The mixture is allowed to stir at 330 rpm under N_2_ overnight. The particles are washed with repeated centrifugation-resuspension cycles at 3000 rpm for 4 h. For the seeded growth, the same procedure is used with 500 mL of deionized water, 55 mL of styrene, and 12 mL of seed particle suspension (2 wt %) added to the flask before heating, sparging, and initiation. The resultant particles are washed repeatedly with centrifugation-resuspension cycles at 1500 rpm for 4 h.

#### Inflation Experiments

The composite droplets are brought to inflation conditions with hydrogen peroxide and NaOH, sealed in a flat capillary, observed, and illuminated with a Leica DMI3000 inverted microscope equipped with a 100x oil immersion objective and a Hamamatsu ORCA Flash 4.0 sCMOS camera. Blue light (430–490 nm) from an external metal halide source (Leica EL6000) coupled to the microscope is shone through the 100x and at 100% and is measured to be 20 mW. A new capillary is made for each experiment. Neutral density filters are used to adjust the intensity. The droplets are analyzed with ImageJ and Matlab.

## Supporting Information Available

Video descriptions and supporting figures (PDF)
Supporting videos (ZIP)

## References

[ref1] Buddingh’ B. C.; van Hest J. C. Artificial cells: synthetic compartments with life-like functionality and adaptivity. Accounts of chemical research 2017, 50, 769–777. 10.1021/acs.accounts.6b00512. 28094501 PMC5397886

[ref2] Needleman D.; Dogic Z. Active matter at the interface between materials science and cell biology. Nature reviews materials 2017, 2, 1–14. 10.1038/natrevmats.2017.48.

[ref3] Jiang W.; Wu Z.; Gao Z.; Wan M.; Zhou M.; Mao C.; Shen J. Artificial cells: past, present and future. ACS Nano 2022, 16, 15705–15733. 10.1021/acsnano.2c06104. 36226996

[ref4] Vale R. D. The molecular motor toolbox for intracellular transport. Cell 2003, 112, 467–480. 10.1016/S0092-8674(03)00111-9. 12600311

[ref5] Lerch M. M.; Grinthal A.; Aizenberg J. Homeostasis as inspiration—Toward interactive materials. Adv. Mater. 2020, 32, 1905554 10.1002/adma.201905554. 31922621

[ref6] Merindol R.; Walther A. Materials learning from life: concepts for active, adaptive and autonomous molecular systems. Chem. Soc. Rev. 2017, 46, 5588–5619. 10.1039/C6CS00738D. 28134366

[ref7] Cadart C.; Venkova L.; Recho P.; Lagomarsino M. C.; Piel M. The physics of cell-size regulation across timescales. Nat. Phys. 2019, 15, 993–1004. 10.1038/s41567-019-0629-y.

[ref8] Shields C. W.; Velev O. D. The evolution of active particles: toward externally powered self-propelling and self-reconfiguring particle systems. Chem. 2017, 3, 539–559. 10.1016/j.chempr.2017.09.006.

[ref9] Wang Z.; Mu Y.; Lyu D.; Wu M.; Li J.; Wang Z.; Wang Y. Engineering shapes of active colloids for tunable dynamics. Curr. Opin. Colloid Interface Sci. 2022, 61, 101608 10.1016/j.cocis.2022.101608.

[ref10] Mallory S. A.; Valeriani C.; Cacciuto A. An active approach to colloidal self-assembly. Annu. Rev. Phys. Chem. 2018, 69, 59–79. 10.1146/annurev-physchem-050317-021237. 29106809

[ref11] Zöttl A.; Stark H. Emergent behavior in active colloids. J. Phys.: Condens. Matter 2016, 28, 253001 10.1088/0953-8984/28/25/253001.

[ref12] Wang W.; Duan W.; Ahmed S.; Mallouk T. E.; Sen A. Small power: Autonomous nano-and micromotors propelled by self-generated gradients. Nano Today 2013, 8, 531–554. 10.1016/j.nantod.2013.08.009.

[ref13] Xu T.; Gao W.; Xu L.-P.; Zhang X.; Wang S. Fuel-free synthetic micro-/nanomachines. Adv. Mater. 2017, 29, 1603250 10.1002/adma.201603250. 28026067

[ref14] Gao W.; Wang J. Synthetic micro/nanomotors in drug delivery. Nanoscale 2014, 6, 10486–10494. 10.1039/C4NR03124E. 25096021

[ref15] Babu D.; Katsonis N.; Lancia F.; Plamont R.; Ryabchun A. Motile behaviour of droplets in lipid systems. Nature Reviews Chemistry 2022, 6, 377–388. 10.1038/s41570-022-00392-8. 37117430

[ref16] Boekhoven J.; Hendriksen W. E.; Koper G. J.; Eelkema R.; van Esch J. H. Transient assembly of active materials fueled by a chemical reaction. Science 2015, 349, 1075–1079. 10.1126/science.aac6103. 26339025

[ref17] Van Ravensteijn B. G.; Hendriksen W. E.; Eelkema R.; Van Esch J. H.; Kegel W. K. Fuel-mediated transient clustering of colloidal building blocks. J. Am. Chem. Soc. 2017, 139, 9763–9766. 10.1021/jacs.7b03263. 28671466

[ref18] Yin Y.; Niu L.; Zhu X.; Zhao M.; Zhang Z.; Mann S.; Liang D. Non-equilibrium behaviour in coacervate-based protocells under electric-field-induced excitation. Nat. Commun. 2016, 7, 10658 10.1038/ncomms10658. 26876162 PMC4756681

[ref19] Alvarez L.; Fernandez-Rodriguez M. A.; Alegria A.; Arrese-Igor S.; Zhao K.; Kröger M.; Isa L. Reconfigurable artificial microswimmers with internal feedback. Nat. Commun. 2021, 12, 4762 10.1038/s41467-021-25108-2. 34362934 PMC8346629

[ref20] Sun S.; Li M.; Dong F.; Wang S.; Tian L.; Mann S. Chemical signaling and functional activation in colloidosome-based protocells. Small 2016, 12, 1920–1927. 10.1002/smll.201600243. 26923794

[ref21] Zhang Y.; Wang Z.; Li M.; Xu C.; Gao N.; Yin Z.; Wang K.; Mann S.; Liu J. Osmotic-Induced Reconfiguration and Activation in Membranized Coacervate-Based Protocells. J. Am. Chem. Soc. 2023, 145, 10396–10403. 10.1021/jacs.3c02540. 37104061

[ref22] Che H.; Cao S.; Van Hest J. C. Feedback-induced temporal control of “breathing” polymersomes to create self-adaptive nanoreactors. J. Am. Chem. Soc. 2018, 140, 5356–5359. 10.1021/jacs.8b02387. 29617118 PMC5920916

[ref23] Cao L.; Chen D.; Caruso R. A. Surface-metastable phase-initiated seeding and Ostwald ripening: a facile fluorine-free process towards spherical fluffy core/shell, yolk/shell, and hollow anatase nanostructures. Angew. Chem., Int. Ed. 2013, 52, 10986–10991. 10.1002/anie.201305819. 24039077

[ref24] Nakata K.; Fujishima A. TiO2 photocatalysis: Design and applications. Journal of photochemistry and photobiology C: Photochemistry Reviews 2012, 13, 169–189. 10.1016/j.jphotochemrev.2012.06.001.

[ref25] Kaur K.; Singh C. V. Amorphous TiO2 as a photocatalyst for hydrogen production: a DFT study of structural and electronic properties. Energy Procedia 2012, 29, 291–299. 10.1016/j.egypro.2012.09.035.

[ref26] Zou J.; Gao J.; Xie F. An amorphous TiO2 sol sensitized with H2O2 with the enhancement of photocatalytic activity. J. Alloys Compd. 2010, 497, 420–427. 10.1016/j.jallcom.2010.03.093.

[ref27] Xu Z.; Hueckel T.; Irvine W. T.; Sacanna S. Transmembrane transport in inorganic colloidal cell-mimics. Nature 2021, 597, 220–224. 10.1038/s41586-021-03774-y. 34497391

[ref28] Silletta E. V.; Xu Z.; Youssef M.; Sacanna S.; Jerschow A. Monitoring molecular transport across colloidal membranes. J. Phys. Chem. B 2018, 122, 4931–4936. 10.1021/acs.jpcb.8b01638. 29665683

[ref29] Youssef M.; Hueckel T.; Yi G.-R.; Sacanna S. Shape-shifting colloids via stimulated dewetting. Nat. Commun. 2016, 7, 12216 10.1038/ncomms12216. 27426418 PMC4960307

[ref30] Anderson J. L. Colloid transport by interfacial forces. Annu. Rev. Fluid Mech. 1989, 21, 61–99. 10.1146/annurev.fl.21.010189.000425.

[ref31] Epstein I. R.; Pojman J. A. An introduction to nonlinear chemical dynamics: oscillations, waves, patterns, and chaos; Oxford University Press, 1998.

[ref32] Tamate R.; Ueki T.; Yoshida R. Self-beating artificial cells: design of cross-linked polymersomes showing self-oscillating motion. Adv. Mater. 2015, 27, 837–842. 10.1002/adma.201404757. 25504232

[ref33] Cheng G.; Lin C.; Perez-Mercader J. Self-Organizing Microdroplet Protocells Displaying Light-Driven Oscillatory and Morphological Evolution. Small 2021, 17, 2101162 10.1002/smll.202101162. 33977654

[ref34] Aubret A.; Martinet Q.; Palacci J. Metamachines of pluripotent colloids. Nat. Commun. 2021, 12, 6398 10.1038/s41467-021-26699-6. 34737315 PMC8569212

