# Supplementary material for: Light-Triggered Inflation of Microdroplets
Source: Chem Mater. 2024 Apr 12;36(8):3970–5. doi: 10.1021/acs.chemmater.4c00732 (PMC11044266; doi:10.1021/acs.chemmater.4c00732)
Supplement: Supplementary file 1 — cm4c00732_si_002.pdf [file cm4c00732_si_002.pdf]

Supporting Information: Light-triggered inflation of microdroplets

Adam W. Hauser[1,2*], Qintian Zhou[1], Paul M. Chaikin[2*], Stefano Sacanna[1*]

[1]Department of Chemistry, New York University, 29 Washington Place, New York, NY 10003, United States.

[2]Center for Soft Matter Research, Department of Physics, New York University, 726 Broadway Avenue, New York, NY 10003, United States.

*Corresponding author(s). E-mail(s): adam.hauser@nyu.edu; chaikin@nyu.edu; s.sacanna@nyu.edu;

Description of videos

All videos are real-time unless stated in the video title.

0.1 percent H2O2 multicompartment formation 50x real time.mp4 – very slow inflation rate shows multi-compartment formation; light is on at 5 s and remains on

0.5 percent H2O2 20x real time.mp4 – slow inflation rate, large volume change; light is on at 5 s and remains on. Deflation-driven motion can be observed.

5 percent H2O2 pH 11 20x real time.mp4 – fast inflation rate, low average volume change; several droplets show damped re-inflation behavior

deflate fast.mp4 – single droplet example of a fast deflation event

deflate slow.mp4 – single droplet example of a slow deflation event. Deflation-driven motion can be observed.

deflate invert.mp4 – single droplet example of an inversion deflation event

deflate and collect 1.mp4 – slow deflation event in the presence of a low concentration of 800 nm PS colloids that exhibit transient attraction to the chemical gradient released

deflate and collect 2 high particle concentration.mp4 – significantly higher colloidal concentration shows a higher number of colloids attracted to chemical gradient upon deflation; here, some irreversible aggregation occurs between PS colloids and oil droplets before deflation

multicycle DPM 20x real time.mp4 – example of small oil/TiO_2 ratio droplets made with dimethoxy silane; this system exhibits multiple re-inflation cycles at 1% H_2O_2 and pH 12; the light is on at 5 s and remains on

References

[1] Kraft, D.J., De Folter, J.W., Luigjes, B., Castillo, S.I., Sacanna, S., Philipse, A.P., Kegel, W.K.: Conditions for equilibrium solid-stabilized emulsions. The Journal of Physical Chemistry B **114**(32), 10347–10356 (2010)

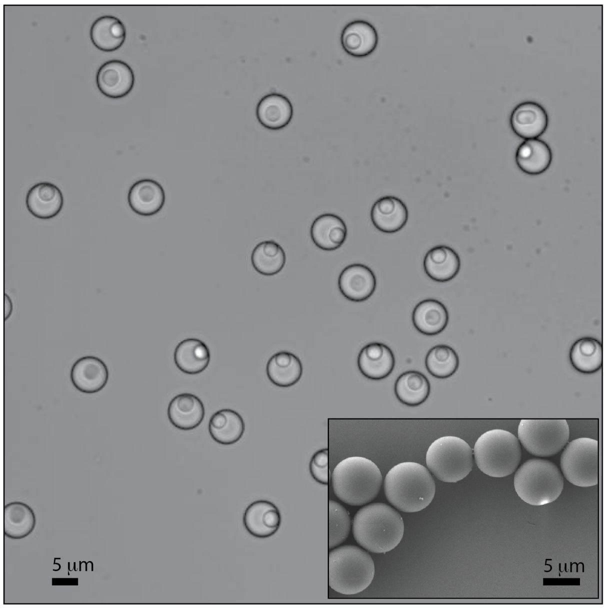

Fig. S1 Optical microscopy image of the composite droplets used in the majority of this study as synthesized in water; inset shows SEM images of polymerized and dried droplets.

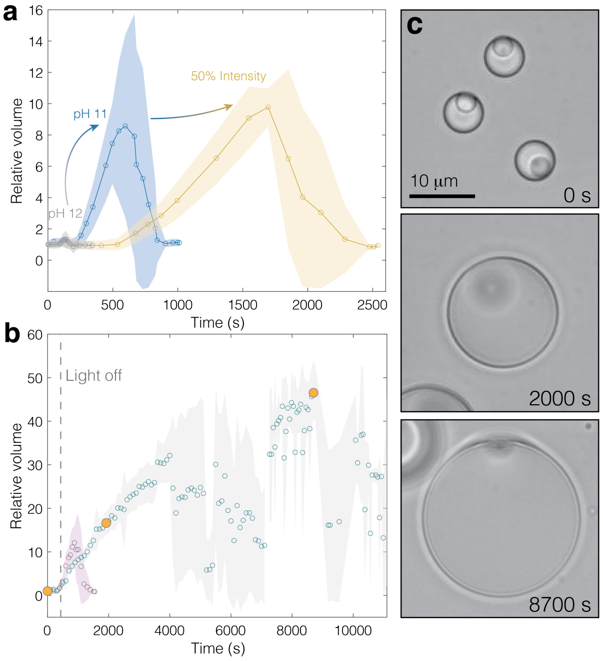

Fig. S2 a) Relative volume vs. time with 10 wt% H_2O_2 in the bulk and constant illumination at pH 12 (gray), pH 11 (blue), and at pH 11 with 50% light intensity (yellow); b) 5 wt% H_2O_2 at pH 11 with constant illumination (purple) and the same sample where the light is turned off at 7 minutes (green); c) snapshots of the slow inflation curve in (b) at the indicated time points.

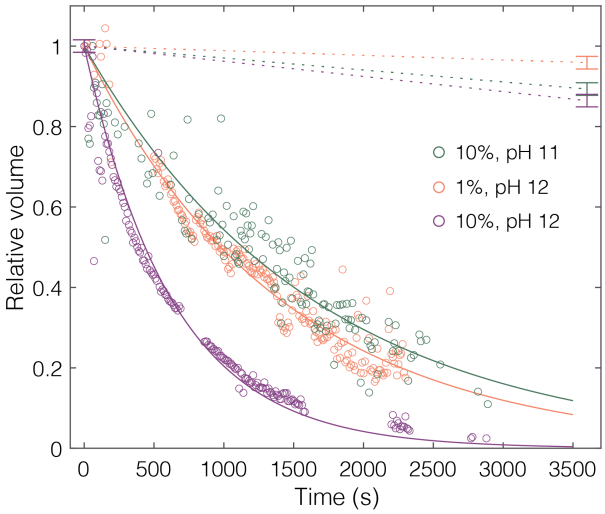

Fig. S3 Relative volume vs. time of the TiO_2 colloids under constant flood illumination at the indicated bulk conditions; dotted lines connect two points in the same samples in the dark showing little volume loss without light.

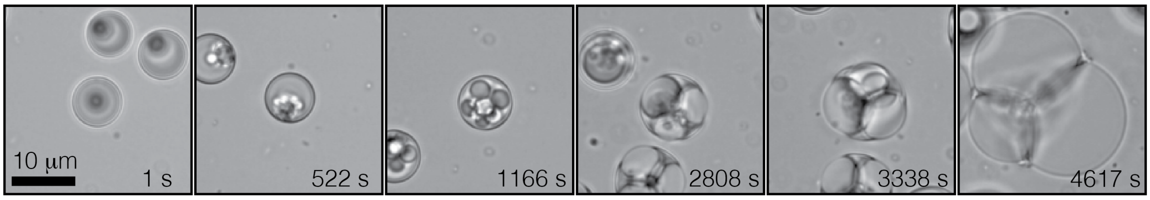

Fig. S4 Timecourse optical microscopy images of inflating droplets under constant illumination at pH 12 and 0.1 wt% H_2O_2 showing very slow inflation relative to Fig. 2a fuel concentrations and the emergence of multi-compartments.

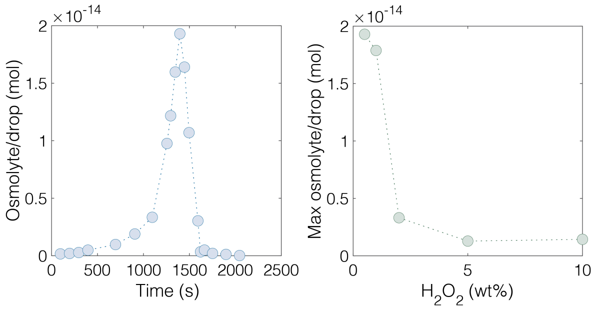

Fig. S5 Estimation of degradants per droplet that create osmotic pressure (osmolyte) vs. time for 0.5 wt% H_2O_2 as in Fig. 2a (left) and estimated maximum osmolyte vs. fuel at pH 12 (right). The internal osmolytes are found from balancing osmotic and Laplace pressure of the droplet: $\Pi - \Pi_e = (C - C_e)RT = 2\gamma(\frac{1}{r_i} + \frac{1}{r_o})$ where Π_e and C_e are external osmotic pressure and external salt concentration (NaOH, 10 mM), Π and C are internal osmotic pressure and osmolyte concentration, R is the gas constant, T is temperature, γ is interfacial tension – assumed to be 10 mN/m at both inner and outer oil-water interfaces [1], and r_o is the measured outer radius while the inner radius is computed assuming oil volume conservation by: $r_i = [\frac{4}{3}\pi(r_o^3 - r_o(0)^3)]^{1/3}$; the y-axes are estimated moles n where $n = C(\frac{4}{3}\pi r_i^3)$.

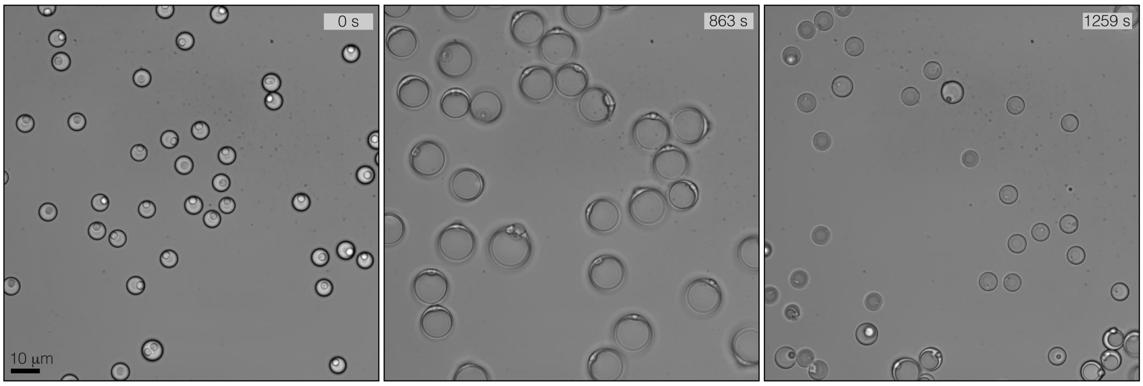

Fig. S6 Optical microscopy images of an example inflation cycle zoomed out to observe the full collected frame; the example here has constant illumination with 1% H_2O_2 at pH 12.

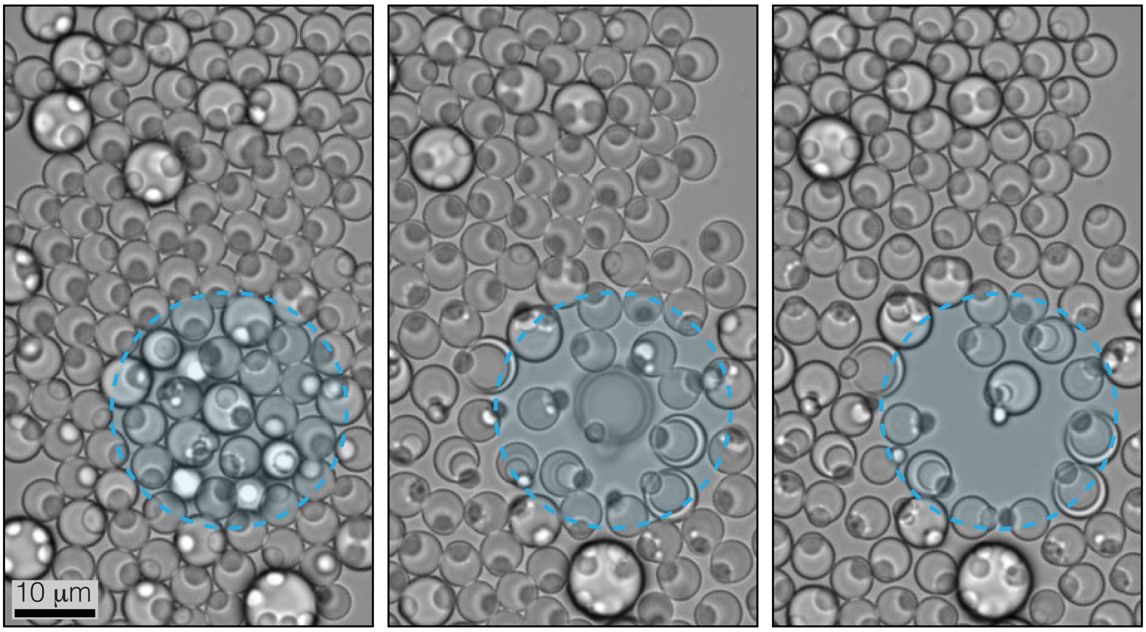

Fig. S7 Optical microscopy images demonstrating spatial selection of inflation in a crowded sample; a small circle is projected, indicated in blue; the time in the light spot from left to right is: 268, 500 and 528 s; the bulk conditions are 5 wt% H_2O_2 at pH 12.

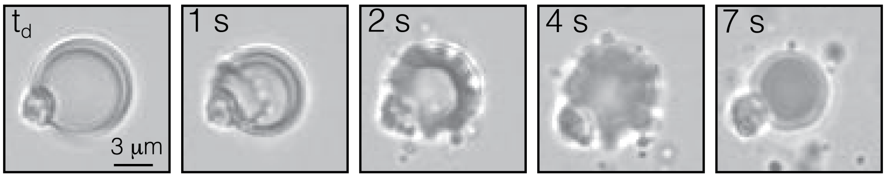

Fig. S8 Inversion deflation type not shown in the main text as it is the rarest observed ($\lesssim$1%); time indicated is relative to when the deflation event begins or t_d.
